# Expression analysis onto microarrays of randomly selected cDNA clones highlights *HOXB13* as a marker of human prostate cancer

**DOI:** 10.1038/sj.bjc.6602261

**Published:** 2004-12-07

**Authors:** S Edwards, C Campbell, P Flohr, J Shipley, I Giddings, R te-Poele, A Dodson, C Foster, J Clark, S Jhavar, G Kovacs, C S Cooper

**Affiliations:** 1 Section of Molecular Carcinogenesis, Male Urological Cancer Research Centre, Institute of Cancer Research, 15 Cotswold Road, Sutton, Surrey SM2 5NG, UK; 2 Department of Engineering Mathematics, University of Bristol, Bristol BS8 1TR, UK; 3 CRUK Centre for Cancer Therapeutics, Male Urological Cancer Research Centre, Institute of Cancer Research, 15 Cotswold Road, Sutton, Surrey SM2 5NG, UK; 4 Department of Pathology & Molecular Genetics, University of Liverpool, Duncan Building, Daulby Street, Liverpool L69 3GA, UK; 5 Section of Cancer Genetics, Male Urological Cancer Research Centre, Institute of Cancer Research, 15 Cotswold Road, Sutton, Surrey SM2 5NG, UK; 6 Laboratory of Molecular Oncology, University Surgical Hospital, Im Neuenheimer Feld 365, Heidelberg 69120, Germany

**Keywords:** prostate, expression, HOXB13, microarray

## Abstract

In a strategy aimed at identifying novel markers of human prostate cancer, we performed expression analysis using microarrays of clones randomly selected from a cDNA library prepared from the LNCaP prostate cancer cell line. Comparisons of expression profiles in primary human prostate cancer, adjacent normal prostate tissue, and a selection of other (nonprostate) normal human tissues, led to the identification of a set of clones that were judged as the best candidate markers of normal and/or malignant prostate tissue. DNA sequencing of the selected clones revealed that they included 10 genes that had previously been established as prostate markers: *NKX3.1*, *KLK2*, *KLK3 (PSA)*, *FOLH1 (PSMA)*, *STEAP2*, *PSGR*, *PRAC*, *RDH11*, *Prostein* and *FASN*. Following analysis of the expression patterns of all selected and sequenced genes through interrogation of SAGE databases, a further three genes from our clone set, *HOXB13*, *SPON2* and *NCAM2*, emerged as additional candidate markers of human prostate cancer. Quantitative RT–PCR demonstrated the specificity of expression of *HOXB13* in prostate tissue and revealed its ubiquitous expression in a series of 37 primary prostate cancers and 20 normal prostates. These results demonstrate the utility of this expression-microarray approach in hunting for new markers of individual human cancer types.

Prostate cancer is a heterogeneous disease with respect to its behaviour and response to hormonal therapy. While some early cancers would be expected to progress rapidly, many cases remain dormant for many years or even decades. A rise in blood PSA level is used widely to detect prostate cancer, but PSA is also elevated in men with chronic prostatitis or benign prostatic hyperplasia (BPH), thus leading to a decrease in the specificity of this marker. Additionally, blood PSA is not significantly increased in some early prostate cancers, particularly those that are poorly differentiated and hence likely to be aggressive. New clinical markers are necessary both to improve specificity of detection and to predict the clinical behaviour of early disease.

Microarray technology has been used to aid the search for new diagnostic and prognostic markers for prostate cancer (Dhanasekaran *et al*, 2001; Luo *et al*, 2001, 2002; Magee *et al*, 2001; Stamey *et al*, 2001; Welsh *et al*, 2001; Ernst *et al*, 2002; LaTulippe *et al*, 2002; Singh *et al*, 2002) (reviewed in Kumar-Sinha *et al*, 2003). These studies have utilised both cDNA and oligonucleotide technologies to examine expression profiles in normal prostate, BPH, prostate cancer and in some cases metastatic hormone-refractory prostatic disease. Previously established markers of prostate cancer such as *KLK2*, *KLK3 (PSA)* and *FOLH1 (PSMA)* were commonly highlighted and an additional cohort of genes consistently upregulated in prostate cancer emerged including *Hepsin*, *AMACR* and *FASN*. Meta-analysis of several of these studies (Rhodes *et al*, 2002) additionally revealed altered transcription of many genes involved in polyamine and purine biosynthesis pathways in prostate cancer. Expression-microarray studies have also identified *EZH2* as a gene overexpressed in hormone-refractory metastatic prostate cancer: notably, patients with clinically localised prostate cancer (Varambally *et al*, 2002) that express EZH2 have a worse prognosis than those that do not express the protein.

In the present study, we have performed expression studies using microarrays of clones randomly selected from cDNA libraries prepared from the LNCaP prostate cancer cell line. The use of a microarray of randomly chosen clones is a crucial first step of selection because the clone set should be enriched in genes overexpressed in prostate cancer. This strategy identified many genes previously confirmed to represent markers of human prostate tissue and highlighted several additional potential markers of this disease, including *HOXB13*, and also offers the prospect of identifying novel genes that may not be preselected for inclusion in a geneset microarray.

## MATERIALS AND METHODS

### Cell lines, tissue procurement and RNA preparation

The LNCaP and PC-3 prostate cancer cell lines and PNT2, an immortalised prostate epithelial cell line, were kindly provided by Professor Norman Maitland and grown in the recommended culture conditions. Total RNA was prepared from these cell lines as described (Wilkinson, 1988). Fresh prostate adenocarcinoma specimens were obtained from men undergoing prostatectomy. Sections from these specimens were used in H&E staining studies to assess the relative proportions of tumour, non-neoplastic epithelium and stroma. Regions of the specimen rich in tumour cells or in non-neoplastic epithelium were selected and preserved in RNAlater® (Ambion, Huntingdon, UK). All tumour specimens chosen for the microarray study were Gleason grade 7 (3+4 or 4+3). Of the additional tumour specimens selected for quantitative PCR analysis (13 TURP, 13 radical prostatectomy), 14 were poorly, 11 moderately and one well differentiated. A further six normal tissues from prostatectomies were also used. RNA from these specimens was prepared using TRIzol® reagent (Invitrogen, Paisley, UK) according to the manufacturer's guidelines with the additional step of a second chloroform extraction before precipitation. RNA from normal prostate and other normal human tissues were obtained from BD Biosciences (Oxford, UK), Ambion (Huntingdon, UK) and Stratagene (La Jolla, CA, USA). The Northern blot was prepared as described before (Clark *et al*, 2002) and probed with the ^32^P-dCTP-labelled PCR-amplified *HOXB13* cDNA clone identified on the microarray.

### cDNA microarrays

A cDNA library from the LNCaP prostate cancer cell line was made using a cDNA synthesis kit (Stratagene, La Jolla, CA, USA) as described previously (Clark *et al*, 2002). Library cDNA clones were picked, grown and stored as described previously (Clark *et al*, 2002). DNA inserts from the picked clones were amplified by PCR and microarrayed onto Gold Seal glass slides (Merck Eurolab Ltd, Poole, UK) coated with poly-L-lysine as described previously (Clark *et al*, 2002). Sample labelling, microarray hybridisation, data collection and data analyses were also carried out as described previously (Clark *et al*, 2002). Hybridisations were performed in triplicate and the results averaged for each clone.

### Ranking of overexpressed genes

To grade the significance in the differences of microarray features between two groups, we initially calculated the ratio of means and applied the Student's *t*-test (Rees, 2001). Two additional tests that are influenced by both the magnitudes and variations of individual features were also applied. These were the Fisher ***F***=(*μ*_1_−*μ*_2_)^2^/(*σ*_1_^2^+*σ*_2_^2^) and Golub ***G***=∣*μ*_1_−*μ*_2_∣/(*σ*_1_−*σ*_2_) score, where *μ*_*i*_ and *σ*_*i*_ are the mean and standard deviation for features belonging to class *i*. In addition to grade features according to the consistency of a difference, the rank-based Mann–Whitney test and the Threshold Number of Misclassifications (TNoM) test were applied (Ben Dor *et al*, 2000; Rees, 2001). For each of the six tests (ratio of means, Student's *t*-test, Mann–Whitney test, TNoM, Fisher score, Golub score), clones with overexpressed patterns in one sample group compared to those observed in a second sample group were ranked according to biggest ratio, most significant difference or most consistent differences. From the rankings derived from these six statistical scores, we also derived an aggregate ranking score. Given the different principles underlying the individual six scores, this aggregate ranking is only loosely defined, but we found this overall score informative (see Results).

### SAGE expression analysis

To examine the breadth of tissue expression of genes represented by the top 100 ranked clones, we mined the summary SAGE expression data from the Cancer Gene Anatomy Project http://cgap.nci.nih.gov/. This method presents the tissue expression level for a representative gene tag sequence by summing the tags for each library of a particular tissue type and normalising to tags/200 000 tags. The best tag for a particular gene was selected by the criteria described http://cgap.nci.nih.gov/SAGE/S AGEHelp. Tissue expression levels for each gene are then depicted by a coloured scale as described http://cgap.nci.nih.gov/Microa rray/MicroArrayHelp. These data also reveal ‘similarly expressed’ genes in the top 100 list as the rows are ordered by strength of correlation.

### Quantitative PCR

cDNA was prepared using Superscript™ II (Invitrogen, Paisley, UK) reverse transcriptase and a random primer (pdN_6_) according to the manufacturer's protocol. The overexpression of *HOXB13* was verified using the TaqMan® quantitative PCR system (Applied Biosystems, Warrington, UK). The Assay-On-Demand™ primer and probe set Hs00197189_m1 (Applied Biosystems, Warrington, UK) was used to specifically detect *HOXB13* transcripts. A *GAPDH* endogenous control reagent (Applied Biosystems) was used as a reference in multiplexed PCR reactions, which were run on an ABI PRISM® 7900HT Sequence Detection System (Applied Biosystems) using TaqMan® Universal PCR Master Mix (Applied Biosystems) with standard thermocycling conditions (50°C 2 min, 95°C 10 min, then 40 cycles of 95°C 15 s, 60°C 1 min). A commercial normal prostate RNA from BD Biosciences was used as the reference (calibrator) sample. The comparative threshold cycle (*C*_T_) method was used for the calculation of fold overexpression where relative amount=2^−ΔΔ*C*_T_^, where −ΔΔ*C*_T_ is the normalised signal level (threshold cycle test gene−threshold cycle endogenous control (*GAPDH*)) in the sample relative to the normalised signal level in the corresponding calibrator sample.

## RESULTS

### Microarray expression profiling

Expression studies were performed using microarrays of 5760 unidentified clones randomly selected from a cDNA library prepared from the LNCaP prostate cancer cell line. In hybridisation studies, 36% of the clones were identified as homologous to mitochondrial sequences and these spots were removed from the microarray electronically prior to data analysis. Microarray expression profiles were collected from 11 prostate cancers, from 10 adjacent regions of morphologically normal prostate and from 12 nonprostate normal tissues (bone marrow, brain, breast, thymus, liver, kidney, skeletal muscle, thyroid, testis, bladder, colon and lung). In each case, the test cDNA labelled with Cy5 was compared to cDNA similarly labelled with Cy3 prepared from the immortalised epithelial prostate cell line PNT2 (Berthon *et al*, 1995).

### Identification of prostate markers

**Figure 1:**
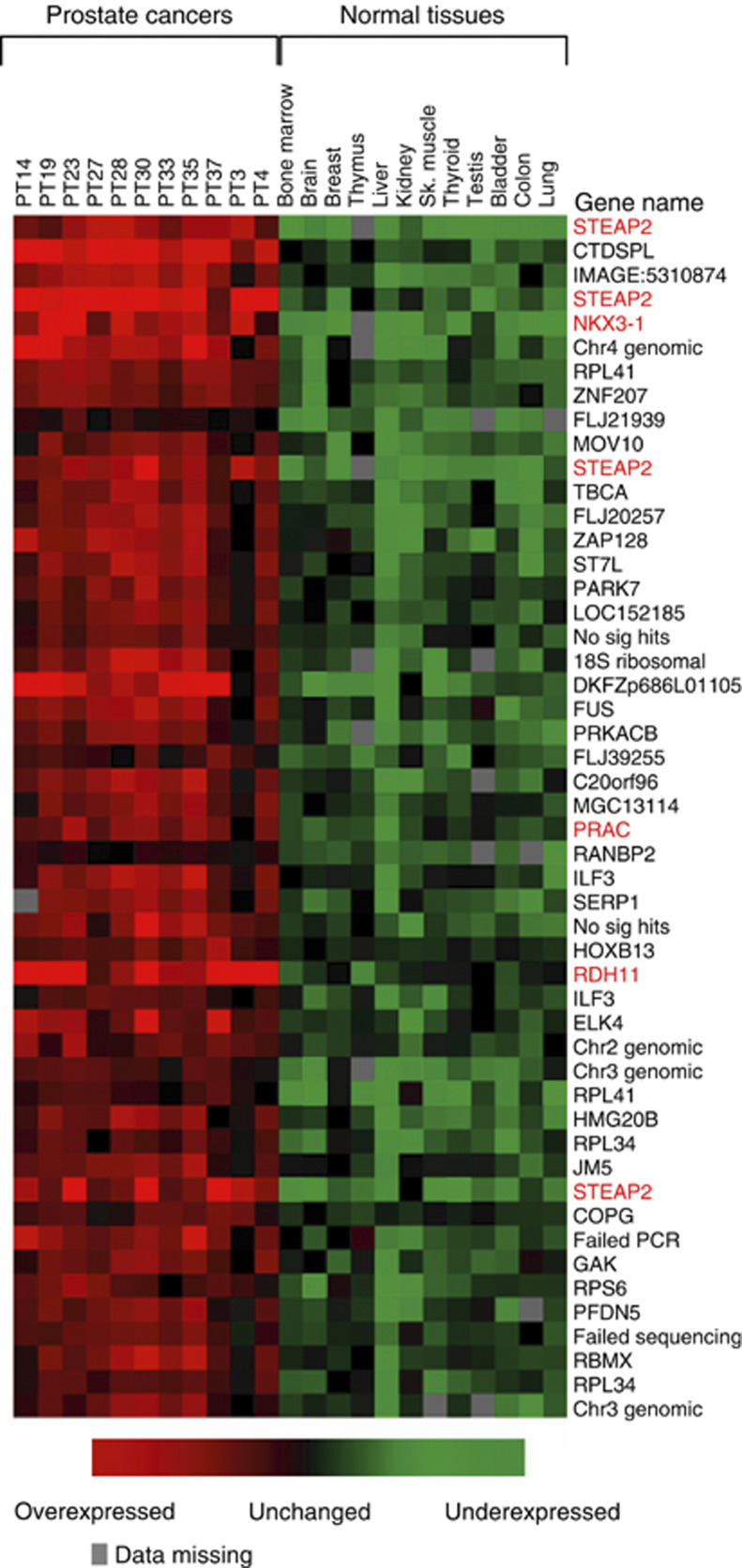
Genes overexpressed in prostate cancer compared to nonprostate normal tissue. We used microarrays of cDNA clones randomly selected from an LNCaP library to identify genes that were overexpressed in a series of 11 prostate cancer samples compared to a series of 12 nonprostate normal tissues. The top 100 ranked overexpressed clones were identified by DNA sequencing. The figure shows a heat map of the expression patterns of the top 50 ranked clones. The full list of 100 clones is available as Supplementary Table 1 (http://www.icr.ac.uk/array/arr ay.html). The red typeface flags genes that have previously been identified as prostate markers.

We first compared the expression of cDNA clones in the 11 prostate cancer samples to their expression in a series of 12 nonprostate normal tissues using the following mathematical tests: ratio of means, Student's *t*-test, Mann–Whitney test, TNoM test, Fisher score and Golub score. The sum of the rankings obtained by each of these tests were then used as a metric to define the overall rank of overexpression for each clone in prostate cancer compared to nonprostate normal tissues. The top 100 clones were then subject to DNA sequencing to reveal their identities (Supplementary Table 1 http://www.icr.ac.uk/array/arr ay.html). Notably, these clones included many genes previously proposed as prostate markers *KLK3 (PSA)*, *PRAC*, *STEAP2* and *RDH11* (Table 1, Figure 1).

The same approach was used to derive a metric that compared expression in the series of 11 samples of morphologically normal prostate tissue collected from regions adjacent to prostate cancer with the same series of 12 nonprostate normal tissues. In all, 20 of the genes previously sequenced were also present in this top 100 list of overexpressed clones (Supplementary Table 2 http://www.icr.ac.uk/array/arr ay.html). The list also included three additional genes, *KLK2*, *PSGR* (*OR51E2*) and *Prostein*, that have previously been established as prostate markers.

The metric was also used to rank the overexpression of clones in 10 prostate cancers compared to the 11 samples of adjacent normal prostate tissue. The top 100 list of overexpressed clones (Supplementary Table 3 http://www.icr.ac.uk/array/arr ay.html) showed a small overlap (six genes) with both the previous two top 100 lists and included two further recognised markers of prostate cancer: *FASN* and *FOLH1 (PSMA)*.

These analyses have highlighted a total of 10 recognised prostate cancer markers, thus providing a strong validation of our approach.

### Novel candidate prostate markers

**Figure 2:**
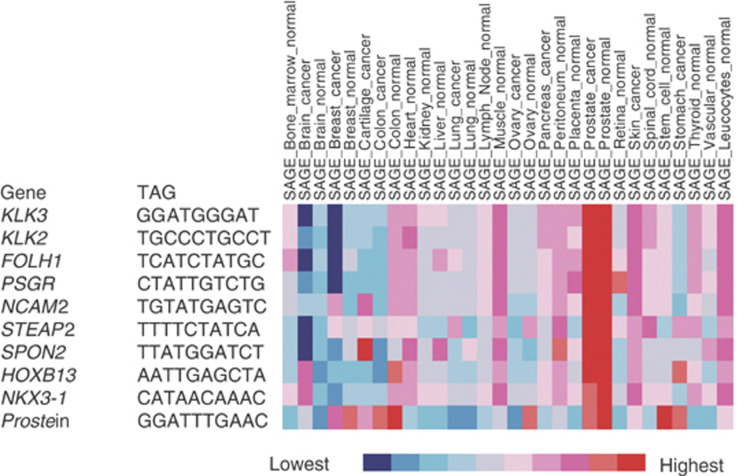
Breadth of gene expression. Gene expression was determined by mining SAGE expression data from the Cancer Genome Anatomy Project (http://cgap.nci.nih.gov). Tissue expression levels for each gene are depicted by a coloured scale as described http://cgap.nci.nih.gov/Microa rray/MicroArrayHelp. The figure shows a group of 10 genes that appear to be highly expressed in the prostate in relation to other cancers and tissues.

To further assess the tissue and tumour specificity of genes in our three top 100 candidate lists, we interrogated SAGE databases for the genes where suitable SAGE tags could be identified. These studies revealed a set of 10 genes that appeared to have higher levels of gene expression in normal and malignant prostate compared to most other normal and cancerous tissue (Figure 2). Other genes in the top 100 lists (Supplementary Tables 1–3) did not appear to exhibit the same specificity for expression in the prostate when subject to SAGE analysis. The list of 10 genes again included the well-established prostate cancer markers *KLK2*, *KLK3 (PSA)*, *FOLH1 (PSMA)*, *PSGR (OR51E2)*, *STEAP2*, *NKX3.1* and *Prostein*), and also included *NCAM2*, *SPON2* and *HOXB13*.

### *NCAM2*, *SPON2* and *HOXB13* as markers of human prostate cancer

*NCAM2* is a close homologue of the neural cell adhesion molecule *NCAM1*. The gene is known to be expressed at high levels in the brain and at low levels in a variety of other adult tissues including the testis, heart and lung (Paoloni-Giacobino *et al*, 1997). *SPON2* was originally isolated as a gene downregulated in lung cancer cell lines compared to normal lung (Manda *et al*, 1999). Initial quantitative RT–PCR experiments (results not shown) demonstrated a highly variable level of expression in prostate cancer with low levels in a range of normal tissue as follows: the thymus, thyroid, breast, colon, liver, lung and bladder. These two genes were not studied further.

**Figure 3:**
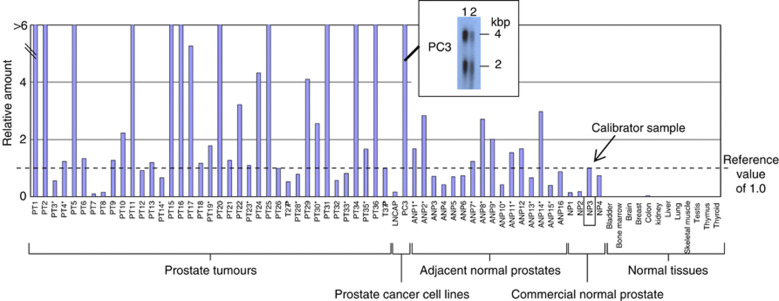
*HOXB13* expression in human tissues. *HOXB13* expression levels were determined by quantitative RT–PCR in a series of 37 primary prostate cancers (PT), 16 samples of morphologically normal prostate tissue taken adjacent to prostate cancer (ANP), four normal prostate samples from individuals who did not have prostate cancer (NP), two prostate cancer cell lines and a selection of nonprostate normal tissues. All amounts are expressed in relation to the levels found in normal prostate sample 3 (NP3). ^*^Samples used in microarray experiments. The inset shows a Northern blot of two independent extractions of PC3 RNA probed with an *HOXB13* cDNA clone confirming the expression of two *HOXB13* transcripts (2 and 4 kbp) in this cell line.

*HOXB13* was present in the top 100 gene lists for all three microarray-based comparisons that we had performed (Supplementary Tables 1–3) and statistical analysis of the microarray data demonstrated that the values in prostate cancer were significantly higher than those found in normal adjacent prostate (Student's *t*-test, *P*<0.001). SAGE database searches indicated that *HOXB13* was expressed in at least two individual primary prostate samples (http://cgap.nci.nih.gov). To establish experimentally that the *HOXB13* gene is specifically upregulated in malignant and normal prostate cancer, we used quantitative RT–PCR to assess levels of *HOXB13* transcripts in 37 primary prostate cancer specimens, 16 samples of adjacent normal prostate, four samples of normal prostate from individuals who did not have prostate cancer and 12 nonprostate normal tissues. Our results show that *HOXB13* transcripts could be detected in all specimens of prostate cancer and in most cases at levels comparable or above those found in normal prostate (Figure 3). Analyses of normal human tissue confirmed that high levels of expression of *HOXB13* is restricted to the prostate, although a low level of *HOXB13* mRNA was also found in the colon.

## DISCUSSION

In a previous study, we have demonstrated that comparative genomic hybridisation and expression studies onto microarrays of clones randomly selected from tumour cell lines containing DNA amplicons are an effective method for identifying candidate amplified and overexpressed genes (Clark *et al*, 2002). In the current study, we have shown that expression studies alone onto microarrays of clones randomly selected from cancer cDNA libraries can also be used to identify tumour markers. The particular design used in this study should enrich the microarray with genes overexpressed in prostate cancer and help avoid exclusion of important genes that can occur in microarrays of preselected genes. Our distinct expression-microarray approach was validated since it identified 10 genes that had previously been established experimentally as prostate markers: *NKX3.1*, *KLK2*, *KLK3 (PSA)*, *PSGR*, *FOLH1 (PSMA)*, *STEAP2*, *PRAC*, *RDH11*, *Prostein* and *FASN*. It is possible that additional markers could have been identified if the number of clones selected from the LNCaP cDNA library had been enlarged. For example, the prostate cancer markers *Hepsin* and *AMACR* are both reported to be expressed in LNCaP cells (Kuefer *et al*, 2002; Srikantan *et al*, 2002) but were not detected as markers in our studies on 5760 clones randomly selected from an LNCaP cDNA library. When our lists of candidate markers were combined with searches of SAGE databases, *HOXB13* emerged as a candidate prostate marker. SAGE database searches indicated that *HOXB13* was expressed in at least two individual primary human prostate samples and in quantitative RT–PCR studies we have experimentally demonstrated for the first time the ubiquitous expression of *HOXB13* in human prostate tissue.

Interestingly *HOXB13* is located at chromosome band 17q21 immediately adjacent to the *PRAC* and *PRAC2* genes, which have also been proposed as markers of human prostate cancer (Liu *et al*, 2001; Olsson *et al*, 2003). It is well established from studies in several eukaryotic species including *Drosophilia* and man that gene expression can be coordinated in domains and the *PRAC-PRAC2-HOXB13* gene cluster may represent a chromosomal domain specifically expressed in prostate.

It is well established that *HOXB13* paralogues are important in prostate development in mice. *HOXA13* and *HOXD13* have roles in the patterning and outgrowth of lobes of the mouse prostate gland (Kondo *et al*, 1997; Podlasek *et al*, 1997, 1999; Warot *et al*, 1997) and *HOXB13* is required for the normal differential and secretory function of mouse prostate (Economides and Capecchi, 2003). *HOXB13* was reported to be expressed only in the prostate and colon of mice in an androgen-independent manner (Sreenath *et al*, 1999) consistent with our expression studies in normal human tissues.

Unlike the human prostate, mouse prostate is divided into four distinct lobes (ventral, dorsal, lateral and anterior). Transgenic mouse studies suggest that expression and function of *HOXB13* is restricted to the mouse ventral prostate (Economides and Capecchi, 2003) where it is involved in controlling secretion of p12, a kazal-type protease inhibitor, and the spermine binding protein p25. Notably, the rodent ventral prostate is not believed to have a counterpart in man (Price, 1963). The observation that *HOXB13* is ubiquitously expressed both in normal and cancerous human prostate could therefore indicate that the distribution of expression of *HOXB13* in mice and man may be distinct, or suggest that the proposition that the ventral mouse prostate does not have a counterpart in man is incorrect.

*NKX3.1*, another homeobox gene involved in the development of mouse prostate, was also highlighted in our studies. *NKX3.1*, unlike *HOXB13*, exhibits androgen-dependent expression (Bieberich *et al*, 1996; Sciavolino *et al*, 1997) and is more broadly expressed in the mouse prostate. *NKX3.1* appears to have a role in prostate cancer development since transgenic mice that have homozygous mutations of the *NKX3.1* gene (Bhatia-Gaur *et al*, 1999; Abdulkadir *et al*, 2002) develop the early cancerous lesion prostatic intraepithelial neoplasia (PIN). Prostatic intraepithelial neoplasia development in these mice is restricted to the anterior mouse prostate lobes and is not found in the ventral prostate lobe. Transgenic mice containing homozygous mutations of the *HOXB13* gene, in contrast, do not appear to be predisposed to prostate cancer development (Economides and Capecchi, 2003). Based on these studies *HOXB13* removal would not be predicted to be involved in prostate cancer development. Recent studies in which overexpression of *HOXB13* was forced in prostate cancer PC3 cells did, however, indicate that this homeodomain protein has the potential to act as a suppressor of cell growth possibly through modulating the expression of TCF-4 (Jung *et al*, 2004). These results, however, conflicted with those obtained by Ma *et al* (2004), who found that HOXB13 enhances mortality and invasion of breast epithelial cells and that the *HOXB13 : IL17BR* expression ratio was predictive of disease-free survival.

In conclusion, we have demonstrated the effectiveness of this expression microarray approach, which utilises microarrays of randomly selected cDNA clones, for identifying markers of individual cancer types. The approach has identified 10 established prostate markers and has further highlighted the potential use of HOXB13 as a diagnostic marker.

## Figures and Tables

**Table 1:** Identification of known prostate-associated genes in microarray-based expression studies

**Unigene ID (build #170)**	**Locus**	**Cancer *vs* tissues (highest rank^a^)**	**Normal *vs* tissues (highest rank^a^)**	**Cancer *vs* normal (highest rank^a^)**	**Reference**
Hs.278695	*Prostein*		18		Xu *et al* (2001)
Hs.181350	*KLK2*		54		Darson *et al* (1997)
Hs.55999	*NKX3.1*	5			Gelmann *et al* (2003)
Hs.83190	*FASN*			6	Welsh *et al* (2001)
Hs.1915	*FOLH1*			67	Burger *et al* (2002)
Hs.408200	*STEAP2*	1	31	4	Korkmaz *et al* (2002)
Hs.116467	*PRAC*	26	78		Liu *et al* (2001)
Hs.171995	*KLK3*	57	3		Stamey *et al* (1987)
Hs.226007	*RDH11*	32	4		Lin *et al* (2001)
Hs.202300	*PSGR (OR51E2)*		19		Xu *et al* (2000)

a Aggregate ranking calculated as described in the Materials and Methods.
