# Supplementary material for: Expression analysis onto microarrays of randomly selected cDNA clones highlights HOXB13 as a marker of human prostate cancer
Source: Br J Cancer. 2004 Dec 7;92(2):376–81. doi: 10.1038/sj.bjc.6602261 (PMC2361840; doi:10.1038/sj.bjc.6602261)
Supplement: Supplementary Table 1 [file 92-6602261x1.doc]

Supplementary Table 1

The top 100 clones distinguishing prostate cancers from non-prostate normal tissues

| Overall  Rank | Sum  of  ranks | Unigene  (Build#170)  or Accession ID | Locus | No. in  class1  (tumours) | No. in  class2 (non-prostate  tissues) | Ratio  of  means | Ratio of means  rank | TNoM score | TNoM  rank | Golub score | Golub  rank | Fisher score | Fisher  rank | M-W  score | M-W  rank | ttest  score | ttest  rank |
| --- | --- | --- | --- | --- | --- | --- | --- | --- | --- | --- | --- | --- | --- | --- | --- | --- | --- |
| 1 | 63 | Hs.376560 | STEAP2 | 11 | 11 | 5.46 | 27 | 0 | 1 | 2.968 | 3 | 13.224 | 1 | 1.45E-03 | 25 | 1.19E-07 | 6 |
| 2 | 65 | Hs.147189 | CTDSPL | 11 | 12 | 4.28 | 45 | 0 | 1 | 2.562 | 5 | 8.920 | 4 | 1.11E-03 | 2 | 2.50E-07 | 8 |
| 3 | 97 | Hs.34656 | IMAGE:  5310874 | 11 | 12 | 3.49 | 56 | 0 | 1 | 1.859 | 16 | 6.413 | 9 | 1.11E-03 | 1 | 6.12E-07 | 14 |
| 4 | 98 | Hs.376560 | STEAP2 | 11 | 12 | 5.69 | 23 | 0 | 1 | 2.239 | 8 | 5.694 | 11 | 1.37E-03 | 18 | 4.13E-06 | 37 |
| 5 | 123 | Hs.55999 | NKX3-1 | 11 | 11 | 5.56 | 26 | 0 | 1 | 2.019 | 9 | 6.711 | 6 | 1.45E-03 | 25 | 9.04E-06 | 56 |
| 6 | 155 |  | genomic 4p15.33 | 11 | 11 | 4.31 | 44 | 0 | 1 | 1.899 | 14 | 5.122 | 12 | 1.81E-03 | 39 | 5.54E-06 | 45 |
| 7 | 171 | Hs.425274 | RPL41 | 11 | 12 | 2.87 | 71 | 0 | 1 | 3.437 | 1 | 11.511 | 2 | 3.13E-03 | 93 | 3.13E-09 | 3 |
| 8 | 180 | Hs.62112 | ZNF207 | 11 | 12 | 2.76 | 80 | 0 | 1 | 3.152 | 2 | 10.611 | 3 | 3.13E-03 | 93 | 5.52E-10 | 1 |
| 9 | 187 | Hs.512689 | AZ2 | 11 | 10 | 2.29 | 118 | 0 | 1 | 1.984 | 12 | 6.493 | 7 | 1.95E-03 | 45 | 2.81E-08 | 4 |
| 10 | 202 | Hs.512586 | MOV10 | 11 | 12 | 2.58 | 88 | 0 | 1 | 1.594 | 43 | 3.705 | 26 | 1.11E-03 | 1 | 4.72E-06 | 43 |
| 11 | 209 | Hs.376560 | STEAP2 | 11 | 11 | 4.62 | 38 | 0 | 1 | 2.855 | 4 | 7.740 | 5 | 4.75E-03 | 148 | 5.79E-07 | 13 |
| 12 | 215 | Hs.24930 | TBCA | 11 | 12 | 2.78 | 77 | 0 | 1 | 1.465 | 64 | 3.723 | 25 | 1.11E-03 | 1 | 5.77E-06 | 47 |
| 13 | 225 | Hs.178011 | FLJ20257 | 11 | 12 | 2.97 | 68 | 1 | 70 | 1.636 | 34 | 4.482 | 15 | 1.23E-03 | 14 | 2.35E-06 | 24 |
| 14 | 243 | Hs.299629 | ZAP128 | 11 | 12 | 3.22 | 60 | 1 | 70 | 1.542 | 48 | 4.255 | 16 | 1.70E-03 | 29 | 1.85E-06 | 20 |
| 15 | 250 | Hs.146513 | ST7L | 11 | 12 | 2.76 | 79 | 0 | 1 | 1.471 | 63 | 3.321 | 43 | 1.11E-03 | 1 | 1.10E-05 | 63 |
| 16 | 252 | Hs.10958 | PARK7 | 11 | 12 | 2.34 | 111 | 0 | 1 | 1.657 | 29 | 3.479 | 35 | 1.37E-03 | 18 | 9.93E-06 | 58 |
| 17 | 258 | Hs.72402 | LOC152185 | 11 | 12 | 2.32 | 114 | 0 | 1 | 1.526 | 50 | 3.352 | 42 | 1.11E-03 | 1 | 7.24E-06 | 50 |
| 18 | 259 |  | n.s.h | 11 | 12 | 2.10 | 149 | 0 | 1 | 1.494 | 59 | 3.627 | 31 | 1.11E-03 | 1 | 1.79E-06 | 18 |
| 19 | 261 |  | 18S ribosomal | 11 | 10 | 5.77 | 61 | 0 | 1 | 1.696 | 24 | 3.630 | 29 | 2.46E-03 | 64 | 2.06E-05 | 82 |
| 19 | 261 | Hs.240443 | DKFZp686L01105 | 11 | 12 | 3.22 | 21 | 0 | 1 | 1.509 | 55 | 3.509 | 34 | 1.11E-03 | 1 | 9.53E-05 | 149 |
| 21 | 271 | Hs.99969 | FUS | 11 | 12 | 2.79 | 76 | 1 | 70 | 1.514 | 53 | 3.919 | 21 | 1.37E-03 | 18 | 3.44E-06 | 33 |
| 22 | 281 | Hs.87773 | PRKACB | 11 | 11 | 2.81 | 75 | 1 | 70 | 1.887 | 15 | 4.497 | 14 | 3.13E-03 | 91 | 1.31E-06 | 16 |
| 23 | 282 |  | Chimeric clone | 11 | 12 | 2.93 | 172 | 0 | 1 | 1.793 | 20 | 3.599 | 32 | 1.70E-03 | 29 | 2.99E-06 | 28 |
| 23 | 282 | Hs.348112 | C20orf96 | 11 | 11 | 2.01 | 69 | 1 | 70 | 1.636 | 35 | 4.050 | 18 | 2.26E-03 | 55 | 3.96E-06 | 35 |
| 25 | 283 | Hs.239500 | MGC13114 | 11 | 12 | 2.32 | 112 | 0 | 1 | 1.501 | 58 | 3.320 | 44 | 1.11E-03 | 4 | 1.16E-05 | 64 |
| 26 | 284 | Hs.116467 | PRAC | 11 | 12 | 2.53 | 89 | 1 | 70 | 1.949 | 13 | 4.118 | 17 | 2.56E-03 | 65 | 3.19E-06 | 30 |
| 27 | 287 | Hs.199179 | RANBP2 | 11 | 10 | 1.86 | 223 | 0 | 1 | 2.390 | 6 | 5.875 | 10 | 1.95E-03 | 45 | 1.45E-09 | 2 |
| 28 | 293 | Hs.256583 | ILF3 | 11 | 12 | 2.59 | 87 | 0 | 1 | 1.651 | 31 | 3.217 | 47 | 2.09E-03 | 48 | 1.66E-05 | 79 |
| 29 | 303 | Hs.76698 | SERP1 | 10 | 12 | 2.84 | 73 | 1 | 70 | 1.646 | 33 | 4.630 | 13 | 3.01E-03 | 89 | 2.35E-06 | 25 |
| 30 | 312 |  | n.s.h | 11 | 12 | 3.16 | 62 | 0 | 1 | 1.457 | 65 | 3.149 | 49 | 1.11E-03 | 5 | 7.52E-05 | 130 |
| 31 | 313 | Hs.66731 | HOXB13 | 11 | 12 | 1.95 | 191 | 0 | 1 | 2.002 | 11 | 3.640 | 27 | 1.11E-03 | 3 | 1.77E-05 | 80 |
| 32 | 331 | Hs.179817 | RDH11 | 11 | 12 | 4.77 | 34 | 0 | 1 | 1.855 | 18 | 3.004 | 54 | 3.13E-03 | 93 | 7.58E-05 | 131 |
| 33 | 342 | Hs.256583 | ILF3 | 11 | 12 | 2.73 | 160 | 1 | 70 | 1.656 | 30 | 3.628 | 30 | 1.88E-03 | 41 | 4.05E-07 | 11 |
| 33 | 342 | Hs.129969 | ELK4 | 11 | 12 | 2.05 | 82 | 0 | 1 | 1.571 | 45 | 2.928 | 59 | 1.70E-03 | 29 | 7.02E-05 | 126 |
| 35 | 346 |  | genomic 2q31.3 | 11 | 12 | 2.13 | 142 | 0 | 1 | 1.702 | 23 | 3.095 | 51 | 2.09E-03 | 48 | 2.01E-05 | 81 |
| 36 | 348 |  | genomic 3q12.3 | 11 | 11 | 2.47 | 97 | 0 | 1 | 2.356 | 7 | 6.486 | 8 | 9.49E-03 | 230 | 2.83E-08 | 5 |
| 37 | 354 | Hs.425274 | RPL41 | 11 | 12 | 2.31 | 116 | 1 | 70 | 1.802 | 19 | 3.988 | 19 | 3.82E-03 | 121 | 2.69E-07 | 9 |
| 38 | 355 | Hs.32317 | HMG20B | 11 | 12 | 2.77 | 78 | 1 | 70 | 1.559 | 46 | 3.272 | 46 | 1.88E-03 | 41 | 1.46E-05 | 74 |
| 39 | 357 | Hs.250895 | RPL34 | 11 | 12 | 2.18 | 129 | 1 | 70 | 1.604 | 42 | 3.423 | 39 | 2.56E-03 | 65 | 4.37E-07 | 12 |
| 39 | 357 | Hs.21753 | JM5 | 11 | 12 | 2.22 | 135 | 0 | 1 | 1.351 | 91 | 3.044 | 53 | 1.11E-03 | 1 | 1.57E-05 | 76 |
| 39 | 357 | Hs.376560 | STEAP2 | 11 | 12 | 4.59 | 39 | 0 | 1 | 1.625 | 37 | 2.902 | 61 | 2.56E-03 | 65 | 1.10E-04 | 154 |
| 42 | 362 | Hs.368056 | COPG | 11 | 12 | 1.83 | 237 | 0 | 1 | 2.016 | 10 | 3.790 | 24 | 1.70E-03 | 29 | 1.03E-05 | 61 |
| 43 | 383 |  | failed to sequence | 11 | 12 | 2.65 | 83 | 1 | 70 | 1.618 | 39 | 3.190 | 48 | 2.56E-03 | 65 | 1.62E-05 | 78 |
| 44 | 393 | Hs.153227 | GAK | 11 | 12 | 2.26 | 124 | 1 | 70 | 1.424 | 70 | 3.069 | 52 | 1.88E-03 | 41 | 4.13E-06 | 36 |
| 45 | 395 | Hs.408073 | RPS6 | 11 | 12 | 2.32 | 113 | 1 | 70 | 1.648 | 32 | 3.387 | 40 | 2.84E-03 | 87 | 8.10E-06 | 53 |
| 46 | 401 | Hs.288856 | PFDN5 | 11 | 11 | 2.40 | 105 | 1 | 70 | 1.487 | 61 | 3.133 | 50 | 2.26E-03 | 55 | 1.00E-05 | 60 |
| 47 | 409 |  | n.s.h | 11 | 12 | 2.46 | 168 | 1 | 70 | 1.503 | 56 | 3.631 | 28 | 2.09E-03 | 48 | 4.52E-06 | 39 |
| 47 | 409 | Hs.380118 | RBMX | 11 | 12 | 2.02 | 98 | 0 | 1 | 1.476 | 62 | 2.561 | 82 | 1.70E-03 | 29 | 8.24E-05 | 137 |
| 49 | 415 | Hs.250895 | RPL34 | 11 | 12 | 2.01 | 173 | 1 | 70 | 1.673 | 27 | 3.316 | 45 | 3.13E-03 | 93 | 1.92E-07 | 7 |
| 50 | 417 |  | genomic 3q25.2 | 11 | 10 | 2.41 | 104 | 0 | 1 | 1.328 | 103 | 2.865 | 64 | 1.95E-03 | 45 | 4.19E-05 | 100 |
| 51 | 420 | AF203815 | alpha gene | 11 | 11 | 7.30 | 10 | 0 | 1 | 1.440 | 67 | 2.522 | 86 | 2.26E-03 | 55 | 2.23E-04 | 201 |
| 52 | 424 | Hs.279607 | LOC401505 | 11 | 12 | 2.06 | 156 | 1 | 70 | 1.369 | 81 | 2.953 | 57 | 1.37E-03 | 18 | 4.72E-06 | 42 |
| 53 | 432 |  | failed to sequence | 11 | 12 | 3.83 | 53 | 1 | 70 | 1.430 | 68 | 2.764 | 70 | 1.70E-03 | 29 | 8.69E-05 | 142 |
| 54 | 436 | Hs.3709 | QP-C | 11 | 12 | 2.37 | 110 | 1 | 70 | 1.693 | 25 | 3.426 | 38 | 5.61E-03 | 159 | 3.87E-06 | 34 |
| 55 | 437 | AF203815 | alpha gene | 11 | 12 | 4.55 | 41 | 1 | 70 | 1.361 | 85 | 2.832 | 66 | 2.56E-03 | 65 | 5.13E-05 | 110 |
| 56 | 443 | Hs.296141 | IMAGE:4249217 | 11 | 11 | 3.33 | 59 | 0 | 1 | 1.357 | 87 | 2.507 | 89 | 1.45E-03 | 25 | 1.65E-04 | 182 |
| 57 | 447 | Hs.296638 | GDF15 | 11 | 12 | 3.84 | 54 | 0 | 1 | 1.397 | 76 | 2.581 | 80 | 1.37E-03 | 18 | 2.61E-04 | 218 |
| 57 | 447 | Hs.171995 | KLK3 | 11 | 12 | 3.69 | 52 | 1 | 70 | 1.667 | 28 | 2.855 | 65 | 3.13E-03 | 93 | 8.37E-05 | 139 |
| 59 | 448 | Hs.139336 | ABCC4 | 11 | 12 | 2.05 | 161 | 0 | 1 | 1.512 | 54 | 2.547 | 84 | 3.13E-03 | 93 | 8.90E-06 | 55 |
| 60 | 450 | AF203815 | alpha gene | 11 | 12 | 4.97 | 29 | 2 | 176 | 1.502 | 57 | 3.441 | 37 | 2.32E-03 | 61 | 2.65E-05 | 90 |
| 61 | 452 |  | genomic 4q24 | 11 | 12 | 2.62 | 86 | 1 | 70 | 1.630 | 36 | 2.907 | 60 | 3.13E-03 | 93 | 4.95E-05 | 107 |
| 62 | 464 | Hs.408073 | RPS6 | 11 | 12 | 2.16 | 140 | 1 | 70 | 1.389 | 78 | 2.800 | 68 | 3.13E-03 | 93 | 1.15E-06 | 15 |
| 63 | 471 |  | n.s.h | 11 | 11 | 2.21 | 133 | 1 | 70 | 1.323 | 104 | 3.001 | 55 | 2.81E-03 | 83 | 2.53E-06 | 26 |
| 64 | 475 |  | failed to sequence | 11 | 6 | 3.04 | 67 | 0 | 1 | 1.857 | 17 | 3.874 | 22 | 1.59E-02 | 302 | 1.18E-05 | 66 |
| 65 | 484 | Hs.8603 | P8 | 11 | 12 | 3.08 | 66 | 0 | 1 | 1.523 | 52 | 2.488 | 93 | 2.09E-03 | 48 | 2.76E-04 | 224 |
| 66 | 488 | Hs.262476 | AMD1 | 11 | 12 | 4.18 | 47 | 0 | 1 | 1.411 | 72 | 2.171 | 116 | 1.70E-03 | 29 | 2.73E-04 | 223 |
| 67 | 490 | Hs.129895 | TBX3 | 11 | 8 | 2.63 | 84 | 0 | 1 | 1.768 | 22 | 3.942 | 20 | 1.32E-02 | 280 | 2.16E-05 | 83 |
| 68 | 494 | Hs.429365 | IMAGE:  6061696 | 11 | 12 | 1.79 | 253 | 1 | 70 | 1.616 | 40 | 2.982 | 56 | 2.56E-03 | 65 | 3.84E-07 | 10 |
| 69 | 497 | Hs.14511 | SCO1 | 11 | 12 | 2.46 | 99 | 1 | 70 | 1.354 | 89 | 2.622 | 76 | 2.56E-03 | 65 | 3.94E-05 | 98 |
| 70 | 508 |  | failed to sequence | 11 | 12 | 2.11 | 147 | 1 | 70 | 1.175 | 159 | 2.689 | 74 | 1.23E-03 | 14 | 4.83E-06 | 44 |
| 71 | 513 | Hs.180414 | HSPA8 | 11 | 12 | 4.78 | 33 | 0 | 1 | 1.608 | 41 | 2.391 | 96 | 5.61E-03 | 159 | 1.69E-04 | 183 |
| 72 | 525 | Hs.48297 | RCHY1 | 10 | 9 | 2.44 | 100 | 1 | 70 | 1.621 | 38 | 3.871 | 23 | 7.05E-03 | 201 | 2.98E-05 | 93 |
| 73 | 547 | Hs.108124 | RPS4X | 11 | 12 | 2.18 | 137 | 1 | 70 | 1.405 | 74 | 2.514 | 88 | 5.61E-03 | 159 | 1.83E-06 | 19 |
| 74 | 548 | Hs.380760 | EHF | 11 | 8 | 2.42 | 101 | 0 | 1 | 1.772 | 21 | 3.383 | 41 | 2.08E-02 | 336 | 5.81E-06 | 48 |
| 75 | 558 | Hs.300141 | RPL39 | 11 | 12 | 2.23 | 128 | 1 | 70 | 1.264 | 120 | 2.769 | 69 | 2.56E-03 | 65 | 4.95E-05 | 106 |
| 76 | 566 | Hs.300141 | RPL39 | 11 | 12 | 2.28 | 120 | 1 | 70 | 1.236 | 134 | 2.712 | 72 | 3.13E-03 | 93 | 1.60E-05 | 77 |
| 77 | 570 | Hs.250895 | RPL34 | 11 | 12 | 1.84 | 230 | 1 | 70 | 1.357 | 86 | 2.379 | 97 | 2.56E-03 | 65 | 2.07E-06 | 22 |
| 78 | 578 | Hs.288856 | PFDN5 | 11 | 12 | 2.48 | 96 | 2 | 176 | 1.346 | 98 | 2.873 | 63 | 2.32E-03 | 61 | 2.27E-05 | 84 |
| 79 | 580 | Hs.296638 | GDF15 | 11 | 12 | 2.63 | 85 | 0 | 1 | 1.371 | 80 | 2.290 | 108 | 1.88E-03 | 41 | 4.50E-04 | 265 |
| 80 | 582 | Hs.285306 | SCLY | 11 | 11 | 2.50 | 91 | 1 | 70 | 1.239 | 130 | 2.456 | 94 | 2.81E-03 | 83 | 5.71E-05 | 114 |
| 81 | 584 | AF203815 | alpha gene | 11 | 12 | 4.51 | 42 | 1 | 70 | 1.302 | 109 | 2.549 | 83 | 3.13E-03 | 93 | 1.79E-04 | 187 |
| 82 | 585 | AF203815 | alpha gene | 11 | 12 | 4.07 | 50 | 1 | 70 | 1.255 | 125 | 2.424 | 95 | 2.56E-03 | 65 | 1.59E-04 | 180 |
| 83 | 586 |  | LOC51084 | 11 | 7 | 1.73 | 282 | 0 | 1 | 1.688 | 26 | 3.527 | 33 | 7.55E-03 | 206 | 4.16E-06 | 38 |
| 84 | 587 |  | failed to sequence | 11 | 11 | 1.97 | 183 | 1 | 70 | 1.350 | 92 | 2.349 | 98 | 2.26E-03 | 55 | 2.57E-05 | 89 |
| 85 | 593 | Hs.372924 | CREB3L4 | 11 | 10 | 2.30 | 117 | 1 | 70 | 1.232 | 136 | 2.940 | 58 | 4.85E-03 | 150 | 1.07E-05 | 62 |
| 86 | 599 | Hs.274416 | NDUFA6 | 10 | 11 | 2.90 | 70 | 1 | 70 | 1.266 | 119 | 2.597 | 78 | 3.10E-03 | 90 | 1.42E-04 | 172 |
| 87 | 600 | Hs.105040 | LOH12CR1 | 11 | 12 | 2.49 | 93 | 1 | 70 | 1.085 | 209 | 2.743 | 71 | 2.56E-03 | 65 | 2.77E-05 | 92 |
| 88 | 615 | Hs.184014 | RPL31 | 11 | 12 | 1.94 | 198 | 1 | 70 | 1.338 | 100 | 2.337 | 99 | 2.32E-03 | 61 | 2.46E-05 | 87 |
| 89 | 617 | Hs.434201 | ZNF11B | 11 | 9 | 2.01 | 171 | 1 | 70 | 1.259 | 122 | 2.804 | 67 | 5.55E-03 | 158 | 3.13E-06 | 29 |
| 90 | 619 | AF203815 | alpha gene | 11 | 12 | 4.10 | 49 | 2 | 176 | 1.276 | 115 | 2.655 | 75 | 3.13E-03 | 93 | 5.35E-05 | 111 |
| 91 | 626 | Hs.408073 | RPS6 | 11 | 12 | 2.03 | 167 | 1 | 70 | 1.320 | 106 | 2.192 | 114 | 3.46E-03 | 115 | 8.57E-06 | 54 |
| 92 | 627 | AF203815 | alpha gene | 11 | 11 | 6.51 | 17 | 1 | 70 | 1.349 | 95 | 2.336 | 100 | 3.48E-03 | 118 | 2.83E-04 | 227 |
| 93 | 630 | Hs.62354 | LRBA | 11 | 12 | 1.69 | 293 | 1 | 70 | 1.255 | 126 | 2.499 | 91 | 1.37E-03 | 18 | 3.37E-06 | 32 |
| 93 | 630 |  | genomic 18q21.31 | 11 | 11 | 3.15 | 63 | 0 | 1 | 1.314 | 107 | 1.939 | 133 | 1.45E-03 | 25 | 6.80E-04 | 301 |
| 95 | 632 | AF203815 | alpha gene | 11 | 12 | 3.85 | 51 | 1 | 70 | 1.378 | 79 | 2.584 | 79 | 4.64E-03 | 143 | 2.44E-04 | 210 |
| 96 | 635 | Hs.75752 | COX7B | 11 | 12 | 2.12 | 146 | 1 | 70 | 1.155 | 167 | 2.182 | 115 | 1.70E-03 | 29 | 5.02E-05 | 108 |
| 97 | 646 |  | failed to PCR | 10 | 8 | 1.66 | 308 | 0 | 1 | 1.368 | 82 | 2.899 | 62 | 5.88E-03 | 176 | 1.46E-06 | 17 |
| 98 | 664 | Hs.177691 | NCAM2 | 8 | 9 | 2.09 | 150 | 0 | 1 | 1.228 | 139 | 3.465 | 36 | 1.24E-02 | 271 | 1.19E-05 | 67 |
| 99 | 673 | Hs.82202 | RPL17 | 11 | 12 | 1.99 | 177 | 1 | 70 | 1.356 | 88 | 2.308 | 105 | 4.21E-03 | 138 | 3.42E-05 | 95 |
| 100 | 689 | Hs.239155 | KCTD3 | 11 | 12 | 1.83 | 235 | 2 | 176 | 1.492 | 60 | 2.529 | 85 | 2.84E-03 | 87 | 5.57E-06 | 46 |

n.s.h – no significant blast hits
