# Supplementary material for: Expression analysis onto microarrays of randomly selected cDNA clones highlights HOXB13 as a marker of human prostate cancer
Source: Br J Cancer. 2004 Dec 7;92(2):376–81. doi: 10.1038/sj.bjc.6602261 (PMC2361840; doi:10.1038/sj.bjc.6602261)
Supplement: Supplementary Table 2 [file 92-6602261x2.doc]

Supplementary Table 2

The top 100 clones distinguishing normal prostate from non-prostate normal tissues

| Overall rank | Sum of ranks | Unigene  /Accession ID | Locus | No. in class1 (normal prostate) | No. in  class2 (Non-prostate normals) | Ratio of means | Ratio of means rank | TNoM score | TNoM rank | Golub score | Golub rank | Fisher score | Fisher rank | M-W score | M-W rank | ttest score | ttest rank |
| --- | --- | --- | --- | --- | --- | --- | --- | --- | --- | --- | --- | --- | --- | --- | --- | --- | --- |
| 1 | 65 | Hs.97128 | ZNF207 | 10 | 12 | 2.34 | 32 | 1 | 6 | 1.585 | 4 | 4.264 | 4 | 8.69E-04 | 15 | 5.11E-05 | 3 |
| 2 | 86 | Hs.180414 | HSPA8 | 10 | 12 | 4.64 | 8 | 1 | 6 | 1.260 | 10 | 1.926 | 25 | 1.00E-04 | 1 | 2.32E-03 | 36 |
| 3 | 110 | Hs.171995 | KLK3 | 9 | 11 | 3.16 | 16 | 1 | 6 | 1.328 | 8 | 2.334 | 18 | 1.24E-03 | 27 | 2.26E-03 | 35 |
| 4 | 165 | Hs.226007 | RDH11 | 10 | 12 | 4.55 | 9 | 1 | 6 | 1.156 | 20 | 1.499 | 51 | 9.78E-04 | 17 | 5.07E-03 | 62 |
| 5 | 174 | Hs.9527 | APR-3 | 9 | 12 | 2.56 | 21 | 2 | 48 | 1.169 | 18 | 1.879 | 27 | 2.90E-04 | 4 | 4.11E-03 | 56 |
| 6 | 182 | Hs.120247 | NUFIP1 | 5 | 12 | 1.56 | 143 | 0 | 1 | 1.780 | 2 | 6.115 | 2 | 1.57E-03 | 30 | 5.24E-05 | 4 |
| 7 | 208 |  | failed to sequence | 10 | 6 | 2.21 | 47 | 1 | 6 | 1.364 | 7 | 2.653 | 10 | 9.24E-03 | 126 | 6.27E-04 | 12 |
| 8 | 223 |  | genomic 6q21 | 4 | 9 | 1.58 | 136 | 0 | 1 | 2.957 | 1 | 14.097 | 1 | 5.48E-03 | 83 | 9.51E-07 | 1 |
| 9 | 231 | Hs.425274 | RPL41 | 10 | 12 | 2.06 | 57 | 2 | 48 | 1.136 | 21 | 1.900 | 26 | 3.01E-03 | 50 | 1.98E-03 | 29 |
| 10 | 238 | Hs.171995 | KLK3 | 10 | 12 | 2.55 | 22 | 2 | 48 | 1.015 | 30 | 1.399 | 63 | 6.06E-04 | 10 | 5.61E-03 | 65 |
| 11 | 242 |  | genomic 9p13.2 | 5 | 11 | 2.02 | 61 | 1 | 6 | 1.195 | 14 | 2.568 | 13 | 1.08E-02 | 137 | 6.19E-04 | 11 |
| 12 | 253 | Hs.19525 | FLJ22794 | 10 | 12 | 1.55 | 147 | 2 | 48 | 1.074 | 25 | 2.262 | 21 | 3.70E-04 | 7 | 2.45E-04 | 5 |
| 13 | 255 | Hs.408073 | RPS6 | 10 | 12 | 1.67 | 105 | 2 | 48 | 0.937 | 48 | 1.751 | 34 | 6.84E-04 | 12 | 4.61E-04 | 8 |
| 14 | 263 | Hs.401929 | RPL10 | 5 | 11 | 1.73 | 86 | 1 | 6 | 1.216 | 13 | 2.636 | 11 | 1.08E-02 | 137 | 5.11E-04 | 10 |
| 15 | 265 | Hs.265174 | RPL32 | 9 | 12 | 1.42 | 244 | 1 | 6 | 1.424 | 6 | 4.038 | 5 | 1.66E-04 | 2 | 7.35E-06 | 2 |
| 16 | 293 | AF203815 | alpha gene | 10 | 12 | 2.42 | 30 | 2 | 48 | 0.928 | 52 | 1.468 | 55 | 3.72E-03 | 59 | 3.36E-03 | 49 |
| 17 | 329 | Hs.288856 | PFDN5 | 10 | 11 | 1.73 | 85 | 2 | 48 | 0.893 | 68 | 1.384 | 64 | 4.91E-04 | 9 | 3.90E-03 | 55 |
| 18 | 333 | Hs.278695 | prostein | 10 | 12 | 5.17 | 7 | 2 | 48 | 0.970 | 41 | 1.071 | 112 | 2.22E-04 | 3 | 1.25E-02 | 122 |
| 19 | 342 | Hs.202300 | OR51E2 | 9 | 12 | 1.67 | 103 | 2 | 48 | 0.960 | 43 | 1.527 | 46 | 2.24E-03 | 38 | 5.53E-03 | 64 |
| 20 | 345 | Hs.324104 | APBA2BP | 8 | 10 | 1.58 | 135 | 2 | 48 | 0.937 | 49 | 1.754 | 32 | 2.92E-03 | 49 | 2.21E-03 | 32 |
| 21 | 355 |  | n.s.h | 4 | 11 | 1.48 | 191 | 1 | 6 | 1.177 | 16 | 2.415 | 15 | 7.44E-03 | 107 | 1.48E-03 | 20 |
| 22 | 357 | AF203815 | alpha gene | 10 | 12 | 3.48 | 12 | 3 | 152 | 0.941 | 47 | 1.504 | 50 | 3.01E-03 | 50 | 3.11E-03 | 46 |
| 23 | 382 | Hs.129895 | TBX3 | 10 | 8 | 2.21 | 48 | 2 | 48 | 0.897 | 63 | 1.354 | 70 | 5.88E-03 | 92 | 4.84E-03 | 61 |
| 24 | 393 | Hs.171995 | KLK3 | 10 | 12 | 2.23 | 46 | 3 | 152 | 0.972 | 38 | 1.306 | 75 | 3.26E-04 | 6 | 6.73E-03 | 76 |
| 25 | 395 |  | genomic 14 | 9 | 10 | 1.74 | 83 | 3 | 152 | 0.983 | 37 | 1.675 | 37 | 1.92E-03 | 32 | 3.73E-03 | 54 |
| 26 | 410 | Hs.129969 | ELK4 | 10 | 12 | 1.98 | 65 | 2 | 48 | 0.896 | 64 | 1.168 | 96 | 2.42E-03 | 39 | 8.76E-03 | 98 |
| 27= | 424 | Hs.158688 | EIF5B | 8 | 4 | 1.37 | 305 | 0 | 1 | 1.589 | 3 | 4.900 | 3 | 6.58E-03 | 96 | 1.28E-03 | 16 |
| 27= | 424 | Hs.408073 | RPS6 | 10 | 12 | 1.78 | 78 | 3 | 152 | 0.920 | 55 | 1.585 | 43 | 4.58E-03 | 72 | 1.73E-03 | 24 |
| 29 | 432 | Hs.9003 | FLJ13868 | 7 | 7 | 1.41 | 255 | 1 | 6 | 1.091 | 23 | 2.381 | 16 | 6.01E-03 | 93 | 2.62E-03 | 39 |
| 30 | 434 | Hs.446628 | RPS4X | 10 | 12 | 1.67 | 104 | 3 | 152 | 0.862 | 76 | 1.485 | 52 | 2.17E-03 | 36 | 1.07E-03 | 14 |
| 31 | 436 | Hs.408200 | STEAP2 | 10 | 12 | 2.14 | 50 | 3 | 152 | 0.896 | 65 | 1.270 | 84 | 7.71E-04 | 13 | 6.21E-03 | 72 |
| 32 | 439 | Hs.374553 | LAMR1 | 5 | 11 | 1.63 | 114 | 1 | 6 | 1.075 | 24 | 2.144 | 22 | 4.62E-03 | 78 | 2.46E-02 | 195 |
| 33 | 441 |  | failed to sequence | 10 | 12 | 1.54 | 150 | 2 | 48 | 0.801 | 106 | 1.263 | 86 | 1.23E-03 | 23 | 1.88E-03 | 28 |
| 34 | 442 | Hs.25367 | RAB4B | 9 | 6 | 1.93 | 67 | 2 | 48 | 0.971 | 39 | 1.697 | 36 | 1.84E-02 | 195 | 4.29E-03 | 57 |
| 35 | 445 | Hs.119598 | RPL3 | 10 | 12 | 1.67 | 106 | 3 | 152 | 0.864 | 75 | 1.463 | 56 | 1.94E-03 | 33 | 1.69E-03 | 23 |
| 36 | 445 |  | failed to sequence | 4 | 5 | 2.53 | 25 | 0 | 1 | 1.446 | 5 | 3.400 | 6 | 1.43E-02 | 159 | 3.75E-02 | 249 |
| 37= | 458 | Hs.178137 | TOB1 | 10 | 12 | 1.35 | 321 | 2 | 48 | 0.989 | 35 | 1.927 | 24 | 1.10E-03 | 21 | 4.76E-04 | 9 |
| 37= | 458 |  | failed to sequence | 7 | 11 | 1.36 | 311 | 1 | 6 | 0.990 | 33 | 1.510 | 49 | 1.11E-03 | 22 | 2.37E-03 | 37 |
| 39 | 460 |  | failed to sequence | 5 | 10 | 1.43 | 238 | 1 | 6 | 0.999 | 32 | 1.756 | 31 | 7.05E-03 | 106 | 3.22E-03 | 47 |
| 40 | 467 | Hs.146170 | CDCP1 | 9 | 11 | 1.49 | 179 | 3 | 152 | 0.933 | 51 | 1.739 | 35 | 2.09E-03 | 35 | 1.11E-03 | 15 |
| 41 | 505 | Hs.441109 | PEX19 | 8 | 6 | 1.37 | 306 | 1 | 6 | 1.227 | 11 | 2.622 | 12 | 4.51E-03 | 71 | 8.83E-03 | 99 |
| 42 | 523 | Hs.275865 | B3GALT4 | 10 | 12 | 1.56 | 142 | 3 | 152 | 0.831 | 89 | 1.376 | 65 | 3.34E-03 | 56 | 1.41E-03 | 19 |
| 43 | 538 | Hs.171995 | KLK3 | 9 | 12 | 3.25 | 15 | 2 | 48 | 0.865 | 74 | 0.918 | 152 | 2.84E-03 | 47 | 2.60E-02 | 202 |
| 44 | 539 | Hs.368056 | COPG | 10 | 12 | 1.34 | 340 | 3 | 152 | 1.163 | 19 | 2.467 | 14 | 4.75E-04 | 8 | 3.43E-04 | 6 |
| 45 | 546 | Hs.171995 | KLK3 | 9 | 12 | 13.15 | 2 | 1 | 6 | 0.893 | 69 | 0.826 | 193 | 2.84E-03 | 47 | 3.31E-02 | 229 |
| 46 | 557 | Hs.147189 | CTDSPL | 10 | 12 | 2.03 | 59 | 3 | 152 | 0.895 | 67 | 1.093 | 106 | 3.72E-03 | 59 | 1.10E-02 | 114 |
| 47 | 562 | Hs.374588 | RPL17 | 10 | 12 | 1.42 | 243 | 1 | 6 | 0.791 | 113 | 1.019 | 120 | 9.78E-04 | 17 | 5.09E-03 | 63 |
| 48 | 567 |  | failed to sequence | 9 | 10 | 1.35 | 324 | 3 | 152 | 1.107 | 22 | 2.364 | 17 | 2.52E-03 | 45 | 3.51E-04 | 7 |
| 49 | 571 | Hs.401929 | RPL10 | 10 | 12 | 1.48 | 186 | 3 | 152 | 0.860 | 77 | 1.362 | 69 | 2.42E-03 | 39 | 3.29E-03 | 48 |
| 50 | 576 | Hs.81008 | FLNB | 10 | 11 | 1.58 | 134 | 3 | 152 | 0.822 | 93 | 1.313 | 74 | 4.85E-03 | 79 | 3.00E-03 | 44 |
| 51 | 579 | Hs.437178 | ACADVL | 10 | 12 | 1.53 | 155 | 3 | 152 | 0.809 | 100 | 1.282 | 80 | 3.01E-03 | 50 | 2.80E-03 | 42 |
| 52 | 584 | Hs.419640 | PARK7 | 10 | 12 | 1.60 | 123 | 3 | 152 | 0.846 | 85 | 1.115 | 102 | 9.78E-04 | 17 | 9.19E-03 | 105 |
| 53 | 585 | Hs.250895 | RPL34 | 8 | 10 | 1.46 | 203 | 3 | 152 | 0.909 | 57 | 1.634 | 41 | 5.13E-03 | 82 | 3.54E-03 | 50 |
| 54 | 594 | Hs.181350 | KLK2 | 10 | 12 | 3.08 | 17 | 2 | 48 | 0.824 | 91 | 0.775 | 220 | 7.71E-04 | 13 | 2.66E-02 | 205 |
| 55 | 596 | Hs.186350 | RPL4 | 10 | 12 | 1.53 | 156 | 3 | 152 | 0.806 | 102 | 1.281 | 81 | 4.13E-03 | 65 | 2.64E-03 | 40 |
| 56 | 597 | Hs.343667 | ELOVL5 | 10 | 12 | 1.79 | 75 | 4 | 361 | 0.927 | 53 | 1.586 | 42 | 2.42E-03 | 39 | 1.87E-03 | 27 |
| 57 | 601 | AF203815 | alpha gene | 10 | 12 | 2.26 | 41 | 3 | 152 | 0.789 | 116 | 1.073 | 110 | 5.62E-03 | 86 | 8.57E-03 | 96 |
| 58 | 610 |  | genomic 13q32.2 | 5 | 12 | 1.46 | 209 | 2 | 48 | 0.915 | 56 | 1.640 | 40 | 1.53E-02 | 168 | 8.23E-03 | 89 |
| 59 | 611 | AF203815 | alpha gene | 10 | 12 | 2.34 | 32 | 3 | 152 | 0.792 | 112 | 1.082 | 109 | 8.35E-03 | 114 | 8.38E-03 | 92 |
| 60 | 616 |  | genomic 3p21.31 | 8 | 12 | 1.34 | 345 | 2 | 48 | 0.907 | 58 | 1.413 | 61 | 5.48E-03 | 83 | 1.63E-03 | 21 |
| 61 | 634 | Hs.239155 | KCTD3 | 6 | 12 | 1.46 | 205 | 3 | 152 | 0.963 | 42 | 1.813 | 29 | 5.73E-03 | 91 | 1.10E-02 | 115 |
| 62= | 643 |  | genomic 3q22.1 | 5 | 9 | 1.70 | 20 | 1 | 6 | 1.013 | 31 | 1.751 | 33 | 3.29E-02 | 281 | 4.31E-02 | 272 |
| 62= | 643 |  | failed to sequence | 10 | 12 | 2.67 | 166 | 3 | 152 | 0.851 | 83 | 1.101 | 104 | 1.55E-03 | 29 | 9.81E-03 | 109 |
| 62= | 643 | Hs.98669 | ADPRHL1 | 6 | 6 | 1.51 | 99 | 2 | 48 | 0.954 | 45 | 1.671 | 38 | 2.50E-02 | 242 | 2.11E-02 | 171 |
| 65 | 649 | Hs.408200 | STEAP2 | 10 | 12 | 2.54 | 23 | 3 | 152 | 0.815 | 97 | 0.852 | 180 | 1.38E-03 | 28 | 2.09E-02 | 169 |
| 66= | 651 | Hs.356794 | RPS24 | 10 | 12 | 1.70 | 98 | 2 | 48 | 0.684 | 191 | 0.893 | 163 | 4.58E-03 | 72 | 6.79E-03 | 79 |
| 66= | 651 |  | genomic 2q31.3 | 9 | 12 | 1.49 | 176 | 3 | 152 | 0.896 | 66 | 1.375 | 66 | 7.70E-03 | 111 | 6.88E-03 | 80 |
| 66= | 651 |  | genomic 17p13.1 | 8 | 12 | 2.08 | 55 | 3 | 152 | 0.853 | 80 | 1.090 | 107 | 3.81E-03 | 63 | 2.45E-02 | 194 |
| 69= | 655 | Hs.254837 | SLC2A4RG | 5 | 11 | 1.29 | 433 | 1 | 6 | 1.169 | 17 | 2.725 | 9 | 7.76E-03 | 113 | 6.76E-03 | 77 |
| 69= | 655 |  | mitochondrial | 4 | 5 | 1.60 | 129 | 1 | 6 | 1.069 | 28 | 2.282 | 20 | 2.75E-02 | 255 | 3.03E-02 | 217 |
| 71 | 672 | Hs.171995 | KLK3 | 10 | 12 | 3.41 | 13 | 2 | 48 | 0.795 | 110 | 0.753 | 232 | 3.72E-03 | 59 | 2.82E-02 | 210 |
| 72= | 678 | Hs.80545 | RPL37 | 10 | 12 | 1.45 | 270 | 3 | 152 | 0.801 | 105 | 1.282 | 79 | 2.42E-03 | 39 | 2.22E-03 | 33 |
| 72= | 678 | Hs.469653 | RPL5 | 10 | 12 | 1.40 | 217 | 3 | 152 | 0.777 | 119 | 1.207 | 93 | 3.72E-03 | 59 | 2.62E-03 | 38 |
| 74 | 681 | Hs.211203 | RBM4 | 8 | 11 | 1.25 | 533 | 2 | 48 | 0.988 | 36 | 1.928 | 23 | 9.57E-04 | 16 | 1.85E-03 | 25 |
| 75 | 682 | Hs.25895 | JFC1 | 5 | 8 | 1.34 | 346 | 2 | 48 | 1.071 | 27 | 2.293 | 19 | 1.28E-02 | 152 | 8.28E-03 | 90 |
| 76 | 688 | Hs.435953 | SPON2 | 9 | 12 | 2.34 | 31 | 3 | 152 | 0.829 | 90 | 0.977 | 131 | 7.70E-03 | 111 | 2.13E-02 | 173 |
| 77 | 689 |  | failed to sequence | 9 | 12 | 2.16 | 49 | 2 | 48 | 0.758 | 131 | 0.793 | 214 | 6.47E-04 | 11 | 3.37E-02 | 236 |
| 78= | 690 | AF203815 | alpha gene | 5 | 11 | 3.84 | 11 | 1 | 6 | 0.971 | 39 | 1.351 | 71 | 2.02E-02 | 204 | 7.51E-02 | 359 |
| 78= | 690 | Hs.116467 | PRAC | 8 | 12 | 1.65 | 110 | 3 | 152 | 0.889 | 70 | 1.299 | 77 | 1.22E-02 | 146 | 1.47E-02 | 135 |
| 80 | 693 | Hs.409634 | RPL18 | 10 | 12 | 1.67 | 107 | 4 | 361 | 0.859 | 78 | 1.345 | 72 | 1.23E-03 | 23 | 3.57E-03 | 52 |
| 81 | 695 | Hs.66731 | HOXB13 | 9 | 12 | 1.33 | 355 | 2 | 48 | 0.956 | 44 | 1.432 | 58 | 6.92E-03 | 104 | 7.39E-03 | 86 |
| 82 | 695 |  | failed to sequence | 7 | 5 | 1.48 | 194 | 2 | 48 | 0.933 | 50 | 1.663 | 39 | 2.30E-02 | 230 | 1.46E-02 | 134 |
| 83 | 717 |  | failed to sequence | 10 | 12 | 1.42 | 246 | 3 | 152 | 0.765 | 125 | 1.171 | 95 | 3.34E-03 | 56 | 2.97E-03 | 43 |
| 84 | 735 | Hs.275865 | B3GALT4 | 10 | 12 | 1.62 | 116 | 4 | 361 | 0.806 | 101 | 1.279 | 83 | 1.94E-03 | 33 | 2.73E-03 | 41 |
| 85 | 736 |  | failed to sequence | 6 | 11 | 1.47 | 200 | 2 | 48 | 0.905 | 60 | 1.536 | 45 | 2.08E-02 | 209 | 2.15E-02 | 174 |
| 86 | 741 | Hs.356794 | RPS24 | 10 | 12 | 1.54 | 151 | 4 | 361 | 0.838 | 87 | 1.369 | 68 | 3.34E-03 | 56 | 1.32E-03 | 18 |
| 87 | 746 |  | failed to sequence | 10 | 12 | 1.52 | 160 | 4 | 361 | 0.898 | 62 | 1.470 | 53 | 6.86E-03 | 97 | 1.06E-03 | 13 |
| 88= | 753 | Hs.425274 | RPL41 | 10 | 12 | 2.24 | 92 | 3 | 152 | 0.705 | 170 | 0.973 | 133 | 9.20E-03 | 122 | 7.33E-03 | 84 |
| 88= | 753 | Hs.159118 | AMD1 | 10 | 12 | 1.71 | 43 | 3 | 152 | 0.761 | 129 | 0.793 | 215 | 1.23E-03 | 23 | 2.40E-02 | 191 |
| 90 | 754 | Hs.408073 | RPS6 | 4 | 11 | 1.72 | 90 | 2 | 48 | 0.874 | 72 | 1.517 | 48 | 3.12E-02 | 269 | 3.28E-02 | 227 |
| 91 | 767 | Hs.374588 | RPL17 | 10 | 12 | 1.48 | 184 | 4 | 361 | 0.836 | 88 | 1.374 | 67 | 3.01E-03 | 50 | 1.31E-03 | 17 |
| 92 | 773 | Hs.171995 | KLK3 | 10 | 11 | 8.73 | 4 | 3 | 152 | 0.823 | 92 | 0.720 | 249 | 3.10E-03 | 55 | 3.13E-02 | 221 |
| 93 | 776 | Hs.256583 | ILF3 | 5 | 12 | 1.41 | 252 | 2 | 48 | 0.874 | 73 | 1.523 | 47 | 1.53E-02 | 168 | 2.36E-02 | 188 |
| 94 | 781 |  | genomic 4p15.33 | 5 | 11 | 2.47 | 28 | 1 | 6 | 0.947 | 46 | 1.405 | 62 | 3.61E-02 | 298 | 6.70E-02 | 341 |
| 95 | 800 |  | failed to sequence | 10 | 12 | 1.69 | 100 | 3 | 152 | 0.717 | 157 | 0.890 | 165 | 6.86E-03 | 97 | 1.43E-02 | 129 |
| 96 | 811 | failed | failed to sequence | 10 | 12 | 1.71 | 93 | 4 | 361 | 0.794 | 111 | 1.240 | 87 | 8.35E-03 | 114 | 3.07E-03 | 45 |
| 97 | 815 | Hs.406534 | HMG20B | 10 | 12 | 1.59 | 131 | 3 | 152 | 0.713 | 163 | 0.884 | 166 | 4.58E-03 | 72 | 1.44E-02 | 131 |
| 98 | 830 |  | mitochondrial | 9 | 12 | 1.60 | 122 | 4 | 361 | 0.803 | 104 | 1.211 | 92 | 4.47E-03 | 70 | 7.15E-03 | 81 |
| 99 | 836 | Hs.425274 | RPL41 | 10 | 11 | 1.72 | 88 | 2 | 48 | 0.644 | 233 | 0.822 | 197 | 1.12E-02 | 145 | 1.27E-02 | 125 |
| 100 | 840 | Hs.171995 | KLK3 | 10 | 8 | 12.03 | 3 | 2 | 48 | 0.762 | 127 | 0.598 | 313 | 4.47E-03 | 69 | 4.56E-02 | 280 |

n.s.h – no significant blast hits
