# Supplementary material for: Expression analysis onto microarrays of randomly selected cDNA clones highlights HOXB13 as a marker of human prostate cancer
Source: Br J Cancer. 2004 Dec 7;92(2):376–81. doi: 10.1038/sj.bjc.6602261 (PMC2361840; doi:10.1038/sj.bjc.6602261)
Supplement: Supplementary Table 3 [file 92-6602261x3.doc]

Supplementary Table 3

The top 100 clones distinguishing prostate cancer from normal prostate tissues

| Overall Rank | Sum of ranks | Unigene/  Accession ID | Locus | no. in class1 (prostate cancers) | no. in class2 (normal prostates) | Ratio of means | Ratio of means rank | TNoM score | TNoM rank | Golub score | Golub rank | Fisher score | Fisher rank | M-W score | M-W rank | ttest score | ttest rank |
| --- | --- | --- | --- | --- | --- | --- | --- | --- | --- | --- | --- | --- | --- | --- | --- | --- | --- |
| 1 | 100 |  | n.s.h | 11 | 7 | 1.97 | 64 | 1 | 10 | 1.855 | 3 | 7.416 | 4 | 6.83E-04 | 17 | 1.67E-06 | 2 |
| 2 | 112 | Hs.296141 | IMAGE:  4249217 | 11 | 5 | 4.04 | 5 | 0 | 1 | 1.704 | 5 | 4.631 | 17 | 1.84E-03 | 74 | 7.98E-05 | 10 |
| 3 | 147 |  | n.s.h | 11 | 5 | 2.67 | 22 | 1 | 10 | 1.661 | 8 | 5.096 | 13 | 1.84E-03 | 74 | 1.36E-04 | 20 |
| 4 | 147 | Hs.408200 | STEAP2 | 11 | 5 | 2.48 | 15 | 0 | 1 | 1.826 | 4 | 7.353 | 5 | 1.84E-03 | 74 | 5.03E-04 | 48 |
| 5 | 181 | Hs.446685 | ZAP128 | 11 | 8 | 2.22 | 38 | 2 | 71 | 1.352 | 18 | 3.965 | 31 | 7.11E-04 | 18 | 4.60E-05 | 5 |
| 6 | 184 | Hs.83190 | FASN | 11 | 10 | 2.35 | 27 | 1 | 10 | 1.146 | 46 | 3.027 | 73 | 5.60E-04 | 16 | 1.01E-04 | 12 |
| 7 | 188 |  | genomic 15q26.1 | 11 | 5 | 2.10 | 51 | 0 | 1 | 1.365 | 17 | 4.874 | 16 | 1.84E-03 | 74 | 1.98E-04 | 29 |
| 8 | 193 |  | n.s.h | 11 | 10 | 1.55 | 173 | 0 | 1 | 1.661 | 9 | 6.723 | 6 | 1.08E-04 | 3 | 9.42E-07 | 1 |
| 9 | 210 |  | failed to sequence | 11 | 10 | 2.11 | 48 | 2 | 71 | 1.262 | 28 | 3.547 | 47 | 4.30E-04 | 10 | 4.94E-05 | 6 |
| 10 | 240 | Hs.406510 | ATP5B | 11 | 10 | 1.49 | 205 | 1 | 10 | 1.565 | 10 | 6.011 | 10 | 1.08E-04 | 2 | 1.38E-05 | 3 |
| 11 | 241 |  | n.s.h | 11 | 10 | 2.07 | 56 | 2 | 71 | 1.232 | 30 | 3.422 | 50 | 9.34E-04 | 30 | 3.78E-05 | 4 |
| 12 | 242 |  | n.s.h | 11 | 8 | 1.86 | 88 | 2 | 71 | 1.294 | 24 | 3.898 | 34 | 7.11E-04 | 18 | 6.36E-05 | 7 |
| 13 | 249 | Hs.239500 | MGC13114 | 11 | 10 | 1.85 | 90 | 2 | 71 | 1.300 | 23 | 3.610 | 43 | 4.91E-04 | 13 | 6.80E-05 | 9 |
| 14 | 256 | Hs.83190 | FASN | 11 | 10 | 2.22 | 39 | 1 | 10 | 1.093 | 60 | 2.750 | 98 | 7.25E-04 | 24 | 1.77E-04 | 25 |
| 15 | 263 |  | genomic 3q27.3 | 11 | 5 | 1.81 | 98 | 1 | 10 | 1.457 | 14 | 5.491 | 11 | 2.68E-03 | 108 | 1.62E-04 | 22 |
| 16 | 276 |  | failed to sequence | 11 | 8 | 1.90 | 74 | 2 | 71 | 1.177 | 40 | 3.339 | 55 | 7.11E-04 | 18 | 1.34E-04 | 18 |
| 17 | 284 | Hs.72402 | LOC152185 | 11 | 9 | 1.89 | 78 | 2 | 71 | 1.202 | 35 | 3.418 | 51 | 9.50E-04 | 38 | 8.54E-05 | 11 |
| 18 | 285 | Hs.98510 | DKFZp686L20145 | 11 | 6 | 1.90 | 75 | 1 | 10 | 1.181 | 38 | 2.748 | 99 | 8.87E-05 | 1 | 6.39E-04 | 62 |
| 19 | 305 | Hs.180414 | HSPA8 | 11 | 10 | 1.78 | 106 | 2 | 71 | 1.180 | 39 | 3.412 | 52 | 7.25E-04 | 24 | 1.05E-04 | 13 |
| 20 | 312 | Hs.459704 | FLJ20444 | 6 | 4 | 2.76 | 13 | 0 | 1 | 3.258 | 1 | 26.725 | 1 | 1.05E-02 | 282 | 1.07E-04 | 14 |
| 21 | 313 | Hs.296638 | GDF15 | 11 | 10 | 2.78 | 12 | 2 | 71 | 1.224 | 31 | 2.465 | 130 | 2.87E-04 | 6 | 6.71E-04 | 63 |
| 22 | 315 |  | 18S ribosomal | 11 | 7 | 1.78 | 107 | 1 | 10 | 1.466 | 13 | 3.923 | 33 | 3.25E-03 | 128 | 1.77E-04 | 24 |
| 23 | 328 | Hs.21753 | JM5 | 11 | 10 | 1.82 | 94 | 2 | 71 | 1.141 | 47 | 2.957 | 76 | 7.25E-04 | 24 | 1.19E-04 | 16 |
| 24 | 336 |  | genomic 3q26.31 | 10 | 6 | 1.82 | 95 | 1 | 10 | 1.343 | 19 | 4.227 | 26 | 4.80E-03 | 163 | 1.77E-04 | 23 |
| 25 | 354 | AF203815 | alpha gene | 11 | 10 | 2.49 | 21 | 2 | 71 | 1.103 | 56 | 2.419 | 137 | 9.34E-04 | 30 | 3.90E-04 | 39 |
| 26 | 367 | Hs.439874 | SERP1 | 10 | 9 | 2.11 | 49 | 2 | 71 | 1.166 | 42 | 3.090 | 71 | 2.52E-03 | 102 | 2.56E-04 | 32 |
| 27 | 374 | Hs.135890 | PF12 | 11 | 5 | 2.14 | 47 | 1 | 10 | 1.194 | 37 | 2.880 | 86 | 3.87E-03 | 142 | 5.50E-04 | 52 |
| 28 | 379 |  | genomic 2q35 | 11 | 8 | 1.74 | 117 | 1 | 10 | 1.107 | 55 | 2.990 | 75 | 1.70E-03 | 69 | 5.53E-04 | 53 |
| 29 | 383 | Hs.83190 | FASN | 11 | 10 | 1.91 | 73 | 1 | 10 | 0.986 | 109 | 2.510 | 126 | 9.34E-04 | 30 | 3.17E-04 | 35 |
| 30 | 395 | Hs.135892 | NCAM2 | 8 | 4 | 1.99 | 62 | 0 | 1 | 1.662 | 7 | 6.637 | 7 | 6.58E-03 | 205 | 1.78E-03 | 113 |
| 31 | 399 | Hs.435065 | RNF3 | 11 | 5 | 1.53 | 184 | 1 | 10 | 1.375 | 16 | 4.392 | 23 | 2.68E-03 | 108 | 6.10E-04 | 58 |
| 32 | 408 | Hs.83190 | FASN | 11 | 10 | 2.29 | 31 | 1 | 10 | 0.961 | 123 | 2.234 | 177 | 7.25E-04 | 24 | 4.41E-04 | 43 |
| 33 | 417 | Hs.355627 | TBCA | 11 | 9 | 1.94 | 68 | 2 | 71 | 1.055 | 73 | 2.608 | 116 | 1.42E-03 | 55 | 2.87E-04 | 34 |
| 34 | 421 | Hs.129903 | POLL | 9 | 5 | 1.93 | 70 | 1 | 10 | 1.422 | 15 | 4.559 | 19 | 6.27E-03 | 200 | 1.67E-03 | 107 |
| 35 | 434 | Hs.26915 | SPTBN2 | 11 | 8 | 1.44 | 256 | 2 | 71 | 1.268 | 26 | 3.890 | 36 | 5.24E-04 | 14 | 2.15E-04 | 31 |
| 36 | 436 | Hs.402697 | EIF4G3 | 11 | 10 | 1.47 | 219 | 1 | 10 | 1.077 | 65 | 2.947 | 77 | 8.23E-04 | 29 | 3.20E-04 | 36 |
| 37 | 437 |  | genomic 12q14.2 | 11 | 10 | 1.45 | 240 | 2 | 71 | 1.204 | 33 | 3.621 | 42 | 7.25E-04 | 24 | 1.85E-04 | 27 |
| 38 | 454 | Hs.180414 | HSPA8 | 11 | 10 | 1.73 | 118 | 3 | 240 | 1.265 | 27 | 3.583 | 45 | 3.29E-04 | 7 | 1.28E-04 | 17 |
| 39 | 461 | Hs.356742 | DRAP1 | 11 | 9 | 1.52 | 188 | 2 | 71 | 1.132 | 50 | 3.277 | 60 | 1.62E-03 | 66 | 1.82E-04 | 26 |
| 40 | 471 |  | failed to sequence | 11 | 6 | 1.42 | 289 | 1 | 10 | 1.330 | 20 | 4.519 | 20 | 2.57E-03 | 104 | 1.97E-04 | 28 |
| 41 | 488 | Hs.240443 | DKFZp686L01105 | 11 | 10 | 2.74 | 14 | 2 | 71 | 1.035 | 80 | 2.019 | 225 | 9.34E-04 | 30 | 7.48E-04 | 68 |
| 42 | 489 | Hs.408200 | STEAP2 | 11 | 10 | 2.23 | 36 | 2 | 71 | 1.016 | 89 | 2.233 | 178 | 1.53E-03 | 56 | 6.10E-04 | 59 |
| 43 | 499 | Hs.127270 | KIAA1545 | 11 | 8 | 1.57 | 163 | 2 | 71 | 1.082 | 62 | 2.906 | 80 | 1.70E-03 | 69 | 5.66E-04 | 54 |
| 44 | 505 | Hs.131736 | NEDD5 | 11 | 7 | 1.43 | 275 | 1 | 10 | 1.140 | 48 | 3.122 | 69 | 9.47E-04 | 37 | 6.95E-04 | 66 |
| 45= | 507 | Hs.95612 | DSC2 | 11 | 5 | 1.91 | 149 | 2 | 71 | 1.007 | 95 | 3.540 | 48 | 1.24E-03 | 53 | 1.32E-03 | 91 |
| 45= | 507 | Hs.178011 | FLJ20257 | 11 | 10 | 1.61 | 72 | 2 | 71 | 0.999 | 103 | 2.289 | 165 | 1.53E-03 | 56 | 4.09E-04 | 40 |
| 47 | 509 | Hs.107720 | FUS | 11 | 10 | 1.86 | 83 | 3 | 240 | 1.128 | 52 | 2.885 | 85 | 9.34E-04 | 30 | 1.35E-04 | 19 |
| 48 | 513 | Hs.97128 | ZNF207 | 11 | 10 | 1.44 | 257 | 2 | 71 | 1.120 | 54 | 3.237 | 64 | 1.20E-03 | 46 | 1.40E-04 | 21 |
| 49 | 518 | Hs.232116 | PRKWNK2 | 11 | 6 | 2.44 | 25 | 1 | 10 | 1.167 | 41 | 2.022 | 222 | 2.57E-03 | 104 | 2.00E-03 | 116 |
| 50 | 539 | Hs.156324 | PRKACB | 11 | 8 | 1.75 | 111 | 3 | 240 | 1.200 | 36 | 3.459 | 49 | 1.28E-03 | 54 | 5.09E-04 | 49 |
| 51 | 542 | Hs.209846 | LRBA | 11 | 9 | 1.44 | 254 | 2 | 71 | 1.039 | 76 | 2.890 | 83 | 7.23E-04 | 21 | 3.48E-04 | 37 |
| 52 | 543 | Hs.153177 | RPS28 | 11 | 5 | 1.67 | 128 | 2 | 71 | 1.223 | 32 | 3.280 | 59 | 5.51E-03 | 182 | 8.03E-04 | 71 |
| 53 | 547 | Hs.405410 | OGT | 11 | 9 | 1.39 | 320 | 1 | 10 | 1.127 | 53 | 3.325 | 56 | 1.62E-03 | 66 | 4.33E-04 | 42 |
| 54 | 548 | Hs.158530 | RAPGEFL1 | 11 | 5 | 1.78 | 105 | 2 | 71 | 1.320 | 21 | 3.256 | 62 | 7.76E-03 | 234 | 5.75E-04 | 55 |
| 55 | 561 | Hs.31074 | SGSH | 11 | 5 | 1.67 | 130 | 1 | 10 | 1.007 | 94 | 3.282 | 58 | 5.51E-03 | 182 | 1.20E-03 | 87 |
| 56 | 569 | Hs.66731 | HOXB13 | 11 | 9 | 1.47 | 215 | 2 | 71 | 1.028 | 84 | 2.625 | 112 | 4.11E-04 | 8 | 1.10E-03 | 79 |
| 57 | 581 |  | genomic 6q23.3 | 11 | 9 | 2.60 | 18 | 2 | 71 | 1.037 | 78 | 1.868 | 264 | 9.50E-04 | 38 | 1.74E-03 | 112 |
| 58 | 584 |  | failed to sequence | 11 | 9 | 1.66 | 131 | 2 | 71 | 1.006 | 96 | 2.306 | 160 | 7.23E-04 | 21 | 1.65E-03 | 105 |
| 59= | 588 |  | failed to sequence | 11 | 8 | 1.99 | 127 | 1 | 10 | 0.963 | 121 | 2.348 | 150 | 2.25E-03 | 91 | 1.27E-03 | 89 |
| 59= | 588 | Hs.408200 | STEAP2 | 11 | 5 | 1.67 | 61 | 1 | 10 | 1.679 | 6 | 6.162 | 9 | 1.84E-03 | 74 | 3.12E-02 | 428 |
| 61 | 592 |  | genomic 5q35 | 10 | 7 | 1.35 | 398 | 1 | 10 | 1.511 | 12 | 5.465 | 12 | 2.11E-03 | 87 | 8.05E-04 | 73 |
| 62 | 601 | Hs.433343 | SRRM2 | 11 | 10 | 1.66 | 135 | 3 | 240 | 1.086 | 61 | 2.829 | 89 | 1.20E-03 | 46 | 2.04E-04 | 30 |
| 63 | 604 | Hs.374588 | RPL17 | 11 | 10 | 1.44 | 249 | 2 | 71 | 1.069 | 67 | 2.743 | 100 | 1.53E-03 | 56 | 6.19E-04 | 61 |
| 64 | 612 | Hs.438037 | GREB1 | 10 | 5 | 2.33 | 28 | 2 | 71 | 1.136 | 49 | 2.336 | 153 | 4.85E-03 | 165 | 3.38E-03 | 146 |
| 65 | 614 | Hs.147189 | CTDSPL | 11 | 10 | 1.71 | 123 | 3 | 240 | 1.067 | 68 | 2.685 | 108 | 9.34E-04 | 30 | 4.67E-04 | 45 |
| 66 | 628 | Hs.433024 | LOC401504 | 11 | 8 | 1.60 | 150 | 2 | 71 | 1.005 | 98 | 2.575 | 120 | 2.95E-03 | 117 | 8.04E-04 | 72 |
| 67 | 641 | Hs.1915 | FOLH1 | 9 | 4 | 2.57 | 19 | 0 | 1 | 0.986 | 110 | 7.753 | 3 | 3.08E-02 | 462 | 4.70E-04 | 46 |
| 68 | 648 | Hs.181368 | PRPF8 | 11 | 5 | 1.56 | 167 | 1 | 10 | 1.203 | 34 | 3.959 | 32 | 3.87E-03 | 142 | 9.83E-03 | 263 |
| 69 | 665 | Hs.300141 | RPL39 | 11 | 10 | 1.77 | 108 | 3 | 240 | 1.057 | 72 | 2.540 | 124 | 2.19E-03 | 88 | 2.84E-04 | 33 |
| 70 | 672 | Hs.356502 | RPLP1 | 11 | 10 | 1.39 | 316 | 2 | 71 | 0.961 | 123 | 2.826 | 91 | 9.34E-04 | 30 | 4.26E-04 | 41 |
| 71 | 679 | Hs.529860 | IMAGE:5229459 | 11 | 6 | 1.36 | 377 | 1 | 10 | 1.101 | 58 | 4.216 | 27 | 1.84E-03 | 73 | 2.79E-03 | 134 |
| 72 | 686 | Hs.272822 | RUVBL1 | 11 | 9 | 1.59 | 158 | 3 | 240 | 1.094 | 59 | 2.687 | 107 | 1.62E-03 | 66 | 6.03E-04 | 56 |
| 73 | 687 |  | mitochondrial | 9 | 6 | 1.37 | 354 | 1 | 10 | 1.058 | 71 | 3.548 | 46 | 2.65E-03 | 107 | 1.52E-03 | 99 |
| 74 | 695 |  | genomic 19p13.3 | 6 | 5 | 1.51 | 191 | 1 | 10 | 1.292 | 25 | 4.324 | 24 | 1.06E-02 | 284 | 4.03E-03 | 161 |
| 75 | 701 | Hs.249798 | NEDD4L | 11 | 10 | 2.48 | 23 | 1 | 10 | 1.032 | 82 | 1.485 | 413 | 1.65E-04 | 4 | 4.28E-03 | 169 |
| 76 | 706 | Hs.512586 | MOV10 | 11 | 10 | 1.78 | 102 | 3 | 240 | 0.997 | 104 | 2.322 | 157 | 1.53E-03 | 56 | 4.80E-04 | 47 |
| 77 | 709 |  | failed to sequence | 10 | 6 | 1.40 | 310 | 2 | 71 | 1.152 | 44 | 3.595 | 44 | 4.80E-03 | 163 | 9.49E-04 | 77 |
| 78 | 714 | Hs.512689 | AZ2 | 11 | 7 | 1.59 | 157 | 2 | 71 | 1.038 | 77 | 2.703 | 106 | 3.25E-03 | 128 | 4.66E-03 | 175 |
| 79 | 735 | Hs.8518 | SEPN1 | 11 | 10 | 1.55 | 171 | 3 | 240 | 1.002 | 102 | 2.827 | 90 | 2.46E-03 | 94 | 3.55E-04 | 38 |
| 80 | 748 | Hs.118483 | MYO6 | 11 | 8 | 2.03 | 58 | 1 | 10 | 1.004 | 99 | 1.612 | 355 | 2.80E-04 | 5 | 6.86E-03 | 221 |
| 81 | 752 | Hs.31532 | LOC92249 | 9 | 4 | 1.39 | 321 | 1 | 10 | 1.544 | 11 | 4.489 | 21 | 1.36E-02 | 320 | 7.55E-04 | 69 |
| 82 | 765 |  | genomic 15q26.1 | 11 | 10 | 1.61 | 147 | 1 | 10 | 0.741 | 309 | 2.501 | 127 | 3.10E-03 | 121 | 5.24E-04 | 51 |
| 83 | 766 | Hs.274416 | NDUFA6 | 10 | 8 | 2.01 | 59 | 3 | 240 | 1.035 | 79 | 2.264 | 170 | 3.37E-03 | 130 | 1.22E-03 | 88 |
| 84 | 767 |  | genomic 19p12 | 11 | 5 | 1.60 | 155 | 2 | 71 | 1.065 | 69 | 3.393 | 54 | 5.51E-03 | 182 | 7.97E-03 | 236 |
| 85 | 769 | AF203815 | alpha gene | 11 | 10 | 2.20 | 41 | 2 | 71 | 0.925 | 146 | 1.647 | 334 | 1.53E-03 | 56 | 2.16E-03 | 121 |
| 86 | 799 | Hs.389777 | PDE9A | 11 | 7 | 1.56 | 165 | 2 | 71 | 0.935 | 142 | 2.421 | 135 | 5.74E-03 | 191 | 1.48E-03 | 95 |
| 87 | 802 | Hs.289093 | RPL35A | 11 | 10 | 1.49 | 200 | 3 | 240 | 1.012 | 91 | 2.547 | 123 | 1.53E-03 | 56 | 1.37E-03 | 92 |
| 88 | 808 | Hs.504892 | ZFP64 | 11 | 9 | 1.78 | 104 | 3 | 240 | 0.963 | 121 | 2.173 | 190 | 2.09E-03 | 86 | 7.26E-04 | 67 |
| 89 | 815 | Hs.288856 | PFDN5 | 11 | 10 | 1.93 | 69 | 3 | 240 | 0.912 | 158 | 2.130 | 197 | 2.46E-03 | 94 | 6.09E-04 | 57 |
| 90 | 833 |  | failed PCR | 6 | 4 | 1.56 | 168 | 0 | 1 | 1.078 | 64 | 4.567 | 18 | 1.05E-02 | 282 | 1.31E-02 | 300 |
| 91 | 836 | Hs.279518 | APLP2 | 11 | 10 | 1.62 | 143 | 3 | 240 | 0.921 | 149 | 2.251 | 172 | 1.53E-03 | 56 | 8.84E-04 | 76 |
| 92 | 847 |  | failed to sequence | 11 | 6 | 4.72 | 2 | 1 | 10 | 0.958 | 126 | 1.438 | 426 | 2.57E-03 | 104 | 4.77E-03 | 179 |
| 93 | 850 |  | n.s.h | 11 | 6 | 1.34 | 406 | 2 | 71 | 1.079 | 63 | 3.401 | 53 | 3.56E-03 | 138 | 2.06E-03 | 119 |
| 94 | 857 | Hs.307915 | ABCC4 | 11 | 9 | 1.53 | 181 | 3 | 240 | 0.983 | 111 | 2.405 | 142 | 9.50E-04 | 38 | 3.34E-03 | 145 |
| 95 | 878 | Hs.459927 | PTMA | 11 | 8 | 1.83 | 93 | 3 | 240 | 0.970 | 116 | 2.086 | 206 | 3.85E-03 | 141 | 1.15E-03 | 82 |
| 96 | 880 | Hs.426930 | ACTB | 11 | 10 | 1.49 | 204 | 2 | 71 | 1.072 | 66 | 2.203 | 183 | 8.27E-03 | 245 | 1.74E-03 | 111 |
| 97 | 883 |  | failed to sequence | 11 | 4 | 1.51 | 195 | 1 | 10 | 0.980 | 112 | 4.136 | 29 | 2.23E-02 | 408 | 2.62E-03 | 129 |
| 98 | 887 | Hs.405917 | FLJ10707 | 11 | 10 | 1.37 | 355 | 3 | 240 | 1.039 | 75 | 2.684 | 109 | 1.06E-03 | 44 | 6.90E-04 | 64 |
| 99 | 891 | Hs.443811 | CALD1 | 11 | 10 | 1.69 | 125 | 3 | 240 | 0.969 | 117 | 1.968 | 236 | 1.20E-03 | 46 | 2.49E-03 | 127 |
| 100 | 892 | Hs.271953 | SESN3 | 8 | 6 | 1.48 | 211 | 2 | 71 | 1.024 | 86 | 2.728 | 102 | 8.13E-03 | 239 | 4.91E-03 | 183 |

n.s.h – no significant blast hits
